# The withdrawal from oncogenetic counselling and testing for hereditary and familial breast and ovarian cancer. A descriptive study of an Italian sample

**DOI:** 10.1186/1756-9966-27-75

**Published:** 2008-11-24

**Authors:** Anita Caruso, Cristina Vigna, Gabriella Maggi, Fabio Massimo Sega, Francesco Cognetti, Antonella Savarese

**Affiliations:** 1 Prevention and Training Activities in Psycho-Oncology, "Regina Elena" National Cancer Institute, Via Elio Chianesi 53, 00144 Rome, Italy; 2 Prevention and Training Activities in Psycho-Oncology, "Regina Elena" National Cancer Institute, Via Elio Chianesi 53, 00144 Rome, Italy; 3 Department of Psycho-Oncology, "Regina Elena" National Cancer Institute, Rome, Via Elio Chianesi, 53, 00144 Rome, Italy; 4 Department of Surgery, "Regina Elena" National Cancer Institute, Via Elio Chianesi 53, 00144, Rome Italy; 5 Department of Medical Oncology, "Regina Elena" Cancer Institute, Rome, Via Elio Chianesi 53, 00144 Rome, Italy; 6 Department of Medical Oncology, "Regina Elena" National Cancer Institute, Via Elio Chianesi 53, 00144 Rome, Italy

## Abstract

**Background:**

Oncogenetic counselling is seldom followed through, even when individuals are eligible according to the test criteria. The basic variables which influence the decision to undergo the genetic counselling process are: risk perception, expected benefit or limitations of genetic testing, general psychological distress or cancer-specific distress, lack of trust in one's emotional reactions when faced with negative events, expected level of family support and communications within the family. The aim of this study was to describe the psychosocial variables of an Italian sample that forgoes genetic counselling.

**Methods:**

From May 2002 to December 2006 a psychological questionnaire was sent out to one hundred and six subjects, who freely requested a first genetic informative consultation, and never asked to have a second visit and the family tree drawn up in order to inquire about their eligibility for genetic testing. Statistical analysis was performed by Pearson chi-square test, t-test and Spearman RHO coefficient.

**Results:**

The survey presents a lack of emotional cohesion and structured roles and rules within the family system and a positive correlation between the number of children, anxiety and risk perception. The main reasons for giving up on counselling were a sense that testing was a waste of time and the inability to emotionally handle the negative consequences of the test outcome. The subjects who maintained that test and an early diagnosis were a "waste of time" experienced more anxiety.

**Conclusion:**

The study revealed the importance to ac knowledging the whole persona and their family system as well as provide information highlighting usefulness of early diagnosis.

## Background

The possibility of taking advantage of genetic counselling for familial/hereditary breast/ovarian cancer enables a woman, with a family history of cancer, to understand their risk of developing the disease during their life time and undertake adequate provision to manage the risk through a programme of clinical observation or eventual precautionary surgery. Furthermore, the possibility of being made aware of one's risk, also enables the counselees to protect their family and in particular their children, involving them in the process of genetic counselling and prevention programs.

A number of studies have reported that counselees claim a great interest in undergoing the genetic test[1-3]; but it is a fact that, in the same studies the number of subjects who actually underwent the genetic test, even if they complied with the eligibility criteria, is decidedly low [4]. The literature concerning the psychological side of onco-genetic counselling often focuses on the variables which influence the decision to undergo the test and show some basic variables such as; estimated cancer risk [2,5], expected benefit or limitations of genetic testing, [6-8], general psychological distress or cancer-specific distress [7,9], lack of trust in one's emotional reactions when faced with negative events[10], expected level of family support and communications within the family[11-13].

The present study aims at describing some psychosocial variables in an Italian group of patients who interrupted the process of genetic counselling for hereditary/familial breast/ovarian cancer, following an initial informative session. Furthermore, it aims at evaluating motivations, reported by the subjects themselves, which have led to their decision to discontinue the process of counselling and probable association between motivation and psychosocial variables.

## Methods

From May 2002 to December 2006, 380 subjects requested a first appointment for genetic counselling at the Department for Hereditary Tumours of the Breast and/or Ovaries of "Regina Elena" National Cancer Institute of Rome. An informative pre-counselling session by the primary care physician is not envisaged, the subjects freely telephone the out-patient office to learn one's risk of developing a breast and/or ovarian tumour. During the initial informative genetic session the oncologist, supported by a psychologist, supplies the patient information concerning BRCA1 and BRCA2 mutation genes which predisposes one to breast/ovarian tumours, concerning the method of transmission, the possibility of prevention and treatment of hereditary pathologies.

The physician offers the counselee to initiate the next appointment in which a family history will be obtained and eligibility for genetic testing will be determined.

Of these 380 subjects who received the first counselling informative session, 106 never asked for a second counselling session to have the family tree drawn up in order to inquire about their eligibility for genetic testing, 28% of the sample.

These 106 subjects were contacted by telephone by a psychologist, in order to request participation in the study. Once permission from patients was obtained, some questionnaires were sent out to be filled in by the counselee and returned in the enclosed stamped envelope provided. This was to be done within 20 days following the first contact, failing which, a second telephone call was made, requesting the return of the questionnaire. The subject was dropped from the study if their questionnaire was not received in forty days. All the procedure was discussed and approved by local Ethic Committee.

### Study Population

From December 2006 to March 2007, 106 subjects, 3 men and 103 women, were contacted. Six subjects refused to take part in the study, 35 did not return the questionnaire, and 9 sent them back incomplete. The study was then carried out on a sample of 56 subjects, 2 men and 54 women, affected by breast and/or ovarian cancer, or healthy but with a family history of cancer (at least one affected relative). 53% took part in the study.

### Instruments

#### Form for the socio-demographic and medical characteristics

The test variables were; age, gender, place of birth, civil status, number of children, education, employment, religion or whether they are practicing, cancer, number of relatives affected by cancer.

#### Cancer Risk Perception (CRP)

One item taken from prior research [14,15], was to evaluate the possible risk of the subject developing cancer. " Mark with a cross on a scale of 0 to 100 what you consider to be your risk of developing, or re-developing, breast and ovarian cancer". Reply was given on a Visual analogue scale of 0 to 100%: 0 representing the lowest risk, 100 the highest and a blank space "do not know".

#### Genetic Risk Perception (GRP)

Another item taken from prior research[16], was to evaluate the likelihood to be a carrier of the BRCA1/BRCA2 genetic mutation. " Mark with a cross on a scale of 0 to 100 what you consider to be your risk of being a carrier of the genetic mutation predisposing one to breast and ovarian cancer". Reply was given on a Visual Analogue Scale from 0 to 100%; 0 representing the lowest risk, 100 the highest and a blank space "do not know".

#### Anxiety and Depression

The Hospital Anxiety and Depression Scale (HADS) [17], Italian version and mean score [18], is used in literature to evaluate the psychological distress in a non-psychiatric setting. It is composed of two scales of 14 items, 7 regarding anxiety and 7 regarding depression. The two scores can be calculated separately with three cut-offs: normal (0–7), borderline (8–10) disturbance (above 11). By calculating the sum of the two scales, it is possible to identify the presence of disturbance in adaptation(cut-off 13–18), or an episode of heavy depression (cut-off > 19). No psychological distress is evidenced if the sum of the two scores totals < 13.

#### Family structure and functioning

Family Adaptability and Cohesion Evaluation Scale (FACES III) of D.H. Olson [19], Italian version by Galimberti C. and Farina M. [20], reliability within this sample α = 0,847, evaluates the perception of family functioning on the basis of adaptability and cohesion according to a Circumplex model developed by David Olson et.al. There are four levels of family cohesion (disengaged, separated, connected and enmeshed), and four levels of adaptability (rigid, structured, flexible and chaotic).

This Circumplex model classifies the family into 16 specific types, or more generally, according to a three-way division defined as " balanced", "mid-range" and "extreme". Moreover, this measure is used to obtain information about the true or ideal functioning of families The discrepancy between the true and ideal family indicates the measure of family dissatisfaction The test is made up of 40 multiple-choice items (20 for the actual family, 20 for the ideal).

#### Motivation

A questionnaire was composed of 22 true/false items to indicate the reasons behind the decision to choose not to pursue genetic counselling. The questionnaire was adapted from prior research to indicate limitations and benefits derived from the test [3] as well as to ascertain possible barriers to undergoing screening, as perceived by women [21].

### Statistics

Descriptive statistics were used to summarize the pertinent information. Association between dichotomous or categorical variables was tested by the Pearson Chi-Square test (for the trend when necessary). Rank-order correlation was measured by the Spearman RHO coefficient. The SPSS (11.0) statistical program was used for analysis.

## Results

### Sample description

The description of the sample is reported in table 1 (socio-demographic and medical characteristics) and in table 2 (psychological characteristics; *14 subjects do not know how to express their risk of breast and/or ovarian cancer and **15 subjects do not know how to express their risk of being carriers of the genetic BRCA1 e BRCA2 mutation genes).

**Table 1:** Socio-demographic and medical characteristics

***N ***= 56 subjects	***Mean***	***Range***
Age	48,6	27–70
Number of relatives affected by cancer	3,9	0–13
	***Frequency***	***%***
**Status**		
Single	17	30,4
Married	39	69,6
**Number of children**		
No children	15	26,8
Up to two children	29	51,8
More than two children	12	21,4
**Education**		
Elementary and middle	19	33,9
Higher	26	46,4
Degree	11	19,6
**Work**		
Workers	27	48,2
Non workers	29	51,8
**Religion**		
Roman Catholic	52	92,9
Other	4	7,1
**Practicing**		
Practicing	28	50
Non practicing	28	50
**Disease**		
Affected	18	32,1
Not affected	38	67,9

**Table 2:** Psychological characteristics

	***Mean***	***Median***	***St Deviation***	***Range***
***Anxiety***	7,9	7,0	3,7	0–16
***Depression***	5,1	4,0	3,5	0–15
***Real cohesion***	39,7	40,0	7,4	11–53
***Real adaptability***	30,0	30,0	7,1	15–50
***Ideal cohesion***	42,1	42,0	6,0	22–50
***Ideal adaptability***	34,5	35,0	5,7	17–46
***Real dissatisfaction***	-2.3	-2.0	8,1	(-25)–(+24)
***Ideal dissatisfaction***	-4.5	-5,0	7,8	(-22)–(+18)
***Number of relatives affected by cancer***	3,9	3,0	3,2	0–13
***Cancer Risk Perception***	38,9	46,0	23,8	0–100*
***Genetic Risk Perception***	39,9	50,0	23,1	0–86.8**

#### Socio-demographic and medical characteristics

The sample was made up of 56 subjects, 2 men and 54 women. Eighteen were affected by breast or ovarian cancer (1 male breast tumour). Thirty-eight were healthy but with a family history of cancer. The average age was 48.6 years and the average number of family members affected by cancer was 3.9.

Most of the sample were Roman Catholic (92%), but only 50% claimed to be practicing, 46% had finished high school, 19% had a university degree and more than a third of the sample had a middle or elementary school level. Seventy percent were married and 73% had at least one child. An equal number of subjects were either unemployed or employed (52%v 48%). There are no significant differences between cancer affected women and healthy women regarding socio-demographic characteristics, risk perception and anxiety levels.

#### Risk Perception of developing breast and ovarian cancer

The mean percentage of risk perception of developing cancer is 49.8%. The score was correlated positively with the number of children (p = 0.01 r = 0.371) and with the level of anxiety (p = 0.07 r = 0.274). 25% of the subjects could give no indication of their risk perception of developing breast/ovarian cancer.

#### Risk perception of being a carrier of the genetic mutation

The mean percentage of risk perception of being a carrier of BRCA1 and BRCA2 mutation shown is 46.7%. this score was positively correlated with the number of children (p > 0.04 r = 0.338) and level of anxiety (p = 0.04 r = 0.332), and 26,7% of subjects could give no indication of their perception of risk of being a carrier of genetic mutation predisposing one to breast/ovarian cancer.

#### Anxiety and Depression

The mean total score obtained from the HAD scale is within the cut off for adaptation disorders (13.9). In 46.4% of subjects there were no signs of anxiety or depression, the rest of the sample showed an equal distribution between a major depressive episode (26.8%) and adaptation disorders (26.8%). In each scale there was an average score of 8.3 (borderline anxiety) and 5.6 (normal depression).

The borderline anxiety level was 30.4%, anxiety disorders 25%, depressive disorders 12.5% and the same percentage of subjects had a borderline level of depression. The score for the single scale of anxiety correlated with the number of children (p = 0.001 r = 0.421).

#### Family Adaptability and Cohesion Evaluation Scale (FACES III)

The average score of true and ideal cohesion were 39.7 (separated families) and 42.1 (connected families), respectively. The mean index of family dissatisfaction for cohesion was -2.35 (negative sign indicates a general tendency for family members to want closer emotional ties). The average of true and ideal adaptability was 30.1 and 34.5 (range of chaotic families for both values) The average index of family dissatisfaction with adaptability was -4.50 (negative score indicates the desire for change towards greater structuring of rules/roles between family members). A third of the families were considered to be extreme 30%, mid-range 59%, and balanced only 11%.

#### Reasons for discontinuing genetic counselling, and associations between socio-demographic, medical and psychological variables. (Table 3)

**Table 3:** Motivation for patient to choose not to pursue genetic counselling

**Reasons for discontinuing genetic counselling**	**Frequency**	**Percentage**
1) The aim of the genetic testing is not clear	10	17,9

2) I do not feel able to face the stress of hospital visits	24	42,9

3) I am afraid of the consequences of any negative information, for me or my family	27	48,2

4) I do not feel it is fair to involve my family	18	32,1

5) I feel guilty towards my family	5	8,9

6) My family is against genetic testing	4	7,1

7) My partner is against genetic testing	3	5,4

8) I do not want to know if I have a genetic mutation which predisposes me to cancer	15	26,8

9) The test is a waste of time as it will not improve the quality of my life	14	25,0

10) The test is a waste of time as I already have cancer	6	10,7

11) The hospital is too far away	21	37,5

12) I do not have time to do all the visits needed for the genetic testing program	19	33,9

13) Having the test will not prevent me from getting cancer	23	41,1

14) I was not able to get an appointment with the doctor because the administration was unavailable	9	16,1

15) I was not able to get an appointment with the doctor because the phone-line was always engaged	13	23,2

16) The doctor was unavailable	2	3,6

17) I did not feel the medical staff was sufficently welcoming	7	12,5

18) I do not feel I can face the stress of having blood tests	2	3,6

19) I would not be able to tell my family any bad news	18	32,1

20) It would be to hard to know that I would probabely develop cancer	24	42,9

21) If I were to be a carrier of a mutation I could not stand the idea of undergoing frequent check-ups	10	17,9

22) I think early diagnosis is a waste of time	4	7,1

One of the reasons given for discontinuing counselling as reported by 48% of the subjects is the fear of the consequences for their family brought about by the results of the genetic test. In particular, 32% believed it was not right to involve their family. The same percentage of subjects, did not feel they could pass on negative information to their family, and 9% of subjects felt guilty towards their family members. Amongst the counselees who claimed their partner to be against genetic testing, 5.4% harboured a level of genetic risk perception, significantly lower (p = 0.002) compared to the rest of the sample. Forty-three percent of counselees showed fear arising from the stress of hospital visits as their reason for discontinuing the counselling, a further 43% the fear of knowing that they had a high risk of developing cancer and 18% felt they would not be able to confront the idea of frequent preventative clinical surveillance if they were found to be mutation carriers. This latter data was reported principally by subjects affected by cancer and answered 'true' to this item, significantly more than healthy subjects(p = 0.047). Thirty four percent of subjects stated that they did not have the time to attend the visits required for counselling, this reason was given by significantly more single, divorced or widowed women compared to married women (p = 0.048). Thirty seven percent claimed that the distance between the hospital and their home was the reason for discontinuing counselling, and 39% held that it was impossible to obtain an appointment for the visits because the phone lines were always busy.

Twelve percent maintained they did not have a good reception from the medical staff, 27% of the subjects had no desire to know whether or not they were predisposed to genetic mutation for breast or ovarian cancer. Amongst these individuals there was an average number of family members affected by cancer, significantly less than those who have a desire to know their likelihood of genetic risk (p = 0.004). Believing the test was a waste of time because it would not halt the disease, was given as the excuse for discontinuing counselling by 41%. This group encountered significantly higher levels of anxiety and depression (p = 0.03; p = 0.02) than the subjects who felt that genetic testing was useful. Among the subjects whose motivation for discontinuing counselling was the idea that genetic investigation does not improve ones quality of life (25%), higher levels of depression were seen compared to the subjects who, on the contrary, believed genetic analysis could improve the quality of life (p = 0.06). Eighteen percent were not sure of the aim of the genetic test, 7% felt that early diagnosis was a "waste of time" and moreover, this group had significantly higher levels of anxiety and depression compared to those who believed early diagnosis to be useful(p = 0.02; p = 0.01). Ten percent of the subjects, felt that genetic testing was of no value as they were already symptomatic.

## Discussion

According to available literature, certain similarities and even novelties in the psychosocial variables emerged in this Italian sample compared to subjects in other countries who take advantage of oncological-genetic counselling. For the risk perception variable, in agreement with previous works, [4,16,22,23], we found a high risk perception of developing a tumour of the breast and/or ovaries and a high risk perception of being carrier of the genetic mutation BRCA1/BRCA2. Our sample showed a similar correlation between anxiety and risk perception found in literature. Literature reports [23,25,26] that a high level of psychological distress combined with high risk perception were both found to be predictors of the decision to undergo the genetic test. Whereas, in our study, the subjects discontinued genetic counselling despite the high level and combination of psychological distress and risk perception. One possible explanation of this discrepancy could be found in the retrospective design of the study. The sometime late request of informations about the discontinuation and assessment of psychological distress could neither represent a punctual picture of the accuracy of the counsellor intervention, nor correlate emotional perturbations at the first step of genetic counselling. However, the discontinuation from genetic counselling in spite of a high risk perception could be also explained by taking into account the difference between general distress and specific cancer distress. In fact, the latter unease about the onset of a tumour would seem to be useful in activating health orientated behaviour [23,26], in particular when associated with a high risk perception. [23,27]. On the contrary, high general distress (measured in this study with HADs) indicated refusal to undergo genetic testing, thereby reflecting and generating fatalism [28] about the future. The possibility that the high level of general distress found in these subjects (disorders of adjustment and borderline anxiety) was one of the obstacles in the decision to continue counselling is corroborated also by the high percentage of the same individuals who felt that genetic testing was "a waste of time" because it would not prevent the onset of the disease or improve the quality of their lives. Of interest, the subjects who have higher levels of anxiety and depression perceived that the testing and early diagnosis is of no use. This was in agreement with the hypothesis in literature that the perceived lack of control over ones health combined with a high level of general distress seems to lead to avoidance or a sense of fatalism towards prevention [4]. As is true for oncological genetic testing, so it is for Huntington's syndrome [29,30], where the awareness of having a mutation is of no help in changing the course of the disease. In the latter case a refusal to undergo genetic testing is often found when the subjects have a high level of general distress caused by anxiety and depression [31]. Often, the possibility of protecting one's children through awareness of their risk and prevention, is a major factor in the decision to undergo genetic testing [2,4,8,10]. Given this, the lack of motivation to undergo genetic testing in this sample may be explained by the perception of not being able to protect their children, as they see it to be useless for cancer prevention. Therefore, it can be hypothesised that a positive correlation between the level of anxiety and number of children may depend on the perception of not being able to protect them. Furthermore, for the subjects who found genetic testing to be of no avail because they were already symptomatic, a lack of knowledge regarding preventive measures for themselves or their family can also be hypothesised. This data would seem to give further support to the continuing attention paid in literature to the necessity of educational sessions of genetic counselling, which highlight the importance of diagnosis and the possibly of adhering to check-up-surveillance programs. A high percentage of subjects feel incapable of handling their emotions regarding any eventual negative information about themselves or their family, the necessity to undergo frequent screening and the knowledge of being at a higher risk of developing breast or ovarian cancer.

The latter data, frequently reported in literature, would confirm that the "anticipated emotional response" seen as unmanageable and insupportable, is one of the most frequent variables hindering the decision to undergo genetic testing. [5,32]. A large percentage of subjects claim they do not have the time to undergo testing, and it is interesting to note that they are mainly women who have reported the loss or absence of a partner (divorced, separate, widows and single). Furthermore, even certain émpasse of an organisational form, such as the distance from the hospital to the home, or busy phone lines seem to have an influence in the discontinuation of counselling, In agreement with literature, the number of family members affected by cancer is of great relevance regarding onco-genetic testing, in fact, the subjects with the least number of affected relatives say they do not wish to know whether or not they have a genetic mutation. Another very relevant variable which has a positive influence over the decision to undergo testing, is being able to count on the support of, and good communication within the family [4,11,24]. It can be hypothesised that the lack of emotional cohesion or structured roles and rules within the family system, seen in this sample, could have a negative influence on the decision to continue with genetic counselling.

## Conclusion

This study showed the importance to take into consideration, during the process of genetic counselling, not only the single variables but also the whole picture gathered from the cognitive and emotional aspects from both the individual and family spheres, in order to assure adequate care of the patient. Moreover, given the evidence in this sample of a lack of knowledge regarding preventive measures for consultants or their family, it appears necessary to provide information highlighting usefulness of early diagnosis, and information regarding how the process of genetic counselling can be helpful for this purpose.

With the aim of verifying the effective importance of each variable in the decision to discontinue genetic counselling, a comparison between subjects who decline or those who wish to continue genetic counselling will be carried out in a further study.

## Competing interests

The authors' declare there no financial competing interests (political, personal, religious, ideological, academic, intellectual, commercial or any other) with the study.  

## Authors' contributions

AC main author project of the study and interpretation of the data, CV patient's data collection, data analysis and interpretation of the data, GM patient's data collection, FS patient's data collection, FC study coordinator, AS project of the study and study coordinator.  
